# Complete Genome Sequence of *Carnobacterium gilichinskyi* Strain WN1359^T^ (DSM 27470^T^)

**DOI:** 10.1128/genomeA.00985-13

**Published:** 2013-11-27

**Authors:** Michael T. Leonard, Nedka Panayotova, William G. Farmerie, Eric W. Triplett, Wayne L. Nicholson

**Affiliations:** Department of Microbiology & Cell Science, University of Florida, Gainesville, Florida, USAa; Interdisciplinary Center for Biotechnology Research, University of Florida, Gainesville, Florida, USAb

## Abstract

We report the complete genome sequence of *Carnobacterium gilichinskyi* strain WN1359, previously isolated from Siberian permafrost and capable of growth under cold (0°C), anoxic, CO_2_-dominated, low-pressure (0.7-kPa) conditions in a simulation of the Mars atmosphere.

## GENOME ANNOUNCEMENT

Recently it was reported that *Carnobacterium* sp. strain WN1359, isolated from Siberian permafrost, was capable of growth under a combination of low-temperature (0°C), low-pressure (0.7-kPa), and CO_2_-enriched anoxic conditions intended to simulate the atmosphere of Mars (1). Based upon cladistic and phenetic analyses, the name *C. gilichinskyi* sp. nov. was proposed, with strain WN1359 being the type strain (W. L. Nicholson, K. Zhalnina, R. R. Oliveira, and E. W. Triplett, submitted for publication). As part of an effort to further investigate the molecular basis of the response of this organism to simulated Martian conditions, we report here the complete genome sequence of the strain.

*C. gilichinskyi* strain WN1359 is from the corresponding author’s strain collection and is deposited as strain DSM 27470^T^ in the German Collection of Microorganisms and Cell Cultures (DSMZ) (http://www.dsmz.de). Its genome was sequenced at the University of Florida Interdisciplinary Center for Biotechnology Research (UF-ICBR) using the PacBio SMRT system (Pacific Biosciences, Menlo Park, CA). A total of 78,692 reads were obtained, with a mean read length of 3,850 bp. The initial PacBio reads were error corrected using the PacBio RS_PreAssembler.1 module with minimum subread length of 500 bp, minimum read quality of 0.80, and minimum seed read length of 6,000 bp. The error correction process yielded 10,736 reads with an average length of 6,421 bp. A single scaffold was assembled directly from the error-corrected reads using Celera assembler (CA) version 7.0 software. The initial genome assembly was further refined using the PacBio RS_Resequencing.1 module with Quiver consensus calling. This process removes sequencing errors remaining in the initial CA assembly and produces the final consensus genome sequence. The chromosome has 2,347,813 bp and an overall GC content of 35.26%. We detected the presence of five plasmids in the genome, consisting of 9,615 bp (designated pWNCR9), 12,656 bp (pWNCR12), 15,476 bp (pWNCR15), 47,068 bp (pWNCR47), and 64,492 bp (pWNCR64). Open reading frame (ORF) prediction and annotation were performed through the Rapid Annotations using Subsystems Technology (RAST) pipeline (2) using GLIMMER (3). Of the 2,152 protein-encoding ORFs present in the circular chromosome, 1,697 (79%) were assigned by similarity to a known annotated protein function, while 455 (21%) were assigned to unknown protein functions. In addition, 1,841 ORFs (86%) were assigned to Clusters of Orthologous Group (COG) categories (4) through the Batch Web CD-Search tool (5). The rRNAs and tRNAs were identified using the “search_for_RNAs” script developed by Niels Larsen (2) and tRNAscan-SE (6), respectively. By these analyses, 74 tRNAs and 8 rRNA operons, comprising 5S, 16S, and 23S rRNA genes, were detected in the genome.

### Nucleotide sequence accession numbers.

The results of this whole-genome shotgun project have been deposited with GenBank under accession numbers CP006812 (chromosome) and CP006813, CP006814, CP006815, CP006816, and CP006817 (plasmids).
